# Onboard early detection and mitigation of lithium plating in fast-charging batteries

**DOI:** 10.1038/s41467-022-33486-4

**Published:** 2022-11-19

**Authors:** Wenxiao Huang, Yusheng Ye, Hao Chen, Rafael A. Vilá, Andrew Xiang, Hongxia Wang, Fang Liu, Zhiao Yu, Jinwei Xu, Zewen Zhang, Rong Xu, Yecun Wu, Lien-Yang Chou, Hansen Wang, Junwei Xu, David Tomas Boyle, Yuzhang Li, Yi Cui

**Affiliations:** 1 Department of Materials Science and Engineering, Stanford University, Stanford, CA 94305 USA; 2 College of Letters and Science, University of California, Berkeley, CA 94720 USA; 3 Department of Chemical Engineering, Stanford University, Stanford, CA 94305 USA; 4 Department of Electrical Engineering, Stanford University, Stanford, CA 94305 USA; 5 SLAC National Accelerator Laboratory, Stanford Institute for Materials and Energy Sciences, Menlo Park, CA 94025 USA; 6 Chemical and Biomolecular Engineering, University of California, Los Angeles, CA 90095 USA

**Keywords:** Batteries, Characterization and analytical techniques, Nanoscience and technology

## Abstract

Fast-charging is considered as one of the most desired features needed for lithium-ion batteries to accelerate the mainstream adoption of electric vehicles. However, current battery charging protocols mainly consist of conservative rate steps to avoid potential hazardous lithium plating and its associated parasitic reactions. A highly sensitive onboard detection method could enable battery fast-charging without reaching the lithium plating regime. Here, we demonstrate a novel differential pressure sensing method to precisely detect the lithium plating event. By measuring the real-time change of cell pressure per unit of charge (dP/dQ) and comparing it with the threshold defined by the maximum of dP/dQ during lithium-ion intercalation into the negative electrode, the onset of lithium plating before its extensive growth can be detected with high precision. In addition, we show that by integrating this differential pressure sensing into the battery management system (BMS), a dynamic self-regulated charging protocol can be realized to effectively extinguish the lithium plating triggered by low temperature (0 °C) while the conventional static charging protocol leads to catastrophic lithium plating at the same condition. We propose that differential pressure sensing could serve as an early nondestructive diagnosis method to guide the development of fast-charging battery technologies.

## Abstract (web summary)

Fast-charging is highly desired for lithium-ion batteries but is hindered by potential hazardous lithium plating and the associated parasitic reactions. Here, the authors report a nondestructive differential pressure sensing method for early detection and mitigation of lithium plating in fast-charging batteries.

## Introduction

Electric vehicles (EVs) are often considered one of the most sustainable forms of transportation, gaining strong support from many governments and leading to fierce competition among automakers^1^. However, EVs still show a long way toward reaching the mainstream audience^2^. According to McKinsey’s consumer survey, range anxiety, the fear that the battery will run out of power before destination, is a significant psychological barrier plaguing EVs’ large-scale adoption^3^. This attitude is based on the common experience that refueling a gas-powered car is within 10 minutes, while comparable EVs’ charging times are usually measured in hours. Fast charging capability is, therefore, a highly desired and favored feature for EV manufacturing to include in their offerings^4^. The United States Department of Energy has also set the goal to enable a 15-minute charging time for high-energy-density lithium-ion batteries (LIBs) with charging power leveling from the current home charger at ~10 kW to 400 kW^5^. However, no EV on the market is capable of accepting such high charging power without obvious parasitic reactions because of current battery technologies.

C-rate, which specifies the inverse time needed to fully charge or discharge a battery at a given current, is used to describe the rate of charge/discharge. State-of-the-art high-energy LIBs generally use graphite as anodes with a low electrochemical potential of 80~200 mV *vs*. Li/Li^+^ within normal cell operation^6,7^. As a primary failure mode of LIBs under fast charging, lithium plating (Li-plating) on the anode significantly sacrifices battery safety, accelerates capacity fade, and deteriorates lifetime. Under strenuous charge conditions of high C-rates (>1 C in this case) and/or low temperatures (0 °C or lower), the strong polarizations, large charge-transfer overpotentials, and/or sluggish kinetics will push the Li^+^ intercalation potential into graphite below the Li/Li^+^ equilibrium potential, which triggers Li-plating in preference to Li^+^ ions intercalation^8^. Li-plating leads to extensive dendrite formation that might penetrate the separator and cause internal short-circuit, resulting in a rapid heat generation or, even worse, thermal runaway and explosion^9,10^. Moreover, electrolyte decomposes upon contact with Li metal, forming excessive solid electrolyte interphases (SEI) and^11^. In addition, partial lithium deposits could electrically/electrochemically disconnect from the anode. Therefore, not all Li deposits can be effectively extracted through discharge, resulting in the formation of “dead Li”. Incessant formation of SEI and “dead Li” consume a significant amount of active Li and available electrolytes, inducing dramatic capacity losses. Fast charging is, therefore, a practice of balancing the ability to charge at high rates without plating lithium. Current battery charging protocols mainly consist of conservative rate steps to avoid potential hazardous Li-plating and associated parasitic reactions due to the lack of reliable and real-time Li-plating detection technology^12,13^. One main reason is that Li-plating exhibits no signal in an easily accessible way, such as voltage and current, in a two-electrode battery system under practical application. Operando detection of Li-plating is thus of great significance for the onboard application to increase safety, extend battery life, and enable fast charging at C-rates commensurate with the electrochemical limits with confidence.

Several nondestructive attempts have been devised to detect Li-plating beyond conventional electrochemical measurements: high precision coulometry is reported to detect Li-plating through the fine decline of Columbic efficiency (CE, the ratio of discharge capacity over charge capacity of a specific electrode in a cell) as a result of the consumption of active lithium ions^14^; microcalorimetry can reveal the signature heat flow of Li-plating^15^; H_2_ monitoring indicates the reaction between deposited lithium and polymer binders^16^. Those strategies demanding expensive and specialized equipment make them more suitable for studies in a laboratory setting and are not compatible with the onboard applications. Abnormal voltage relaxation and differential voltage analysis were also proposed as indicators of the existence of metallic lithium for onboard detection^17–19^. Although these methods do not involve special equipment, they only detect the aftereffects or their accuracy could be falsely affected by a temperature gradient, ionic gradient, or cell degradations. Irreversible thickness change in the cell has been proposed as an indicator of Li-plating but fails to detect the real-time lithium plating event^20,21^. Mechanical detecting methods, such as force-based incremental capacity analysis^22^, and force model analysis^23^, can provide additional signals beyond electrical signals, which hold great potential to monitor battery health but remain underexplored. There is a significant knowledge gap that needs to be addressed before these strategies can be applied in real scenarios. An ideal onboard detection for Li-plating should meet the following criteria: 1. Nondestructive; 2. Be able to detect lithium dendrite growth; 3. Preferably not changing existing cell structure and fabrication; 4. Integration with battery management system (BMS); 5. Accessible. Currently, no detection technique fulfills all these requirements.

**Fig. 1:**
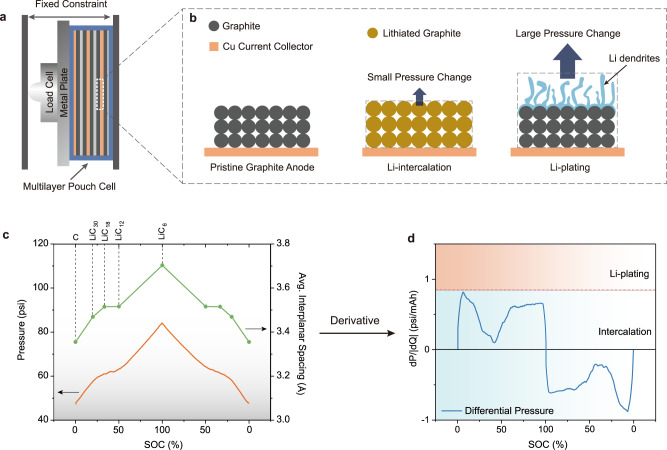
Set-up and the principle of differential pressure sensing for early detection of Li-plating. **a** The configuration of operando pressure measurement. The stack containing a multilayer pouch cell, a metal force-distribution plate, and a pressure sensor (load cell), is clamped onto a mechanically fixed constraint. **b** Zoomed-in schematic of the graphite anode illustrating that Li-plating induces a higher volume/pressure change than intercalation for the same amount of charge passed. **c** The average interplanar lattice spacing of graphite at different lithiation stages agrees with the pressure profile of a 70 mAh NMC-graphite multilayer pouch cell. The cell was charged and discharged at 0.2 C under which Li-plating is unlikely to happen. **d** The differential pressure (dP/dQ) profile of the pouch cell in a full cycle. The red dashed line is the upper bound of dP/dQ during intercalation defining the Li-plating threshold. When Li-plating happens, the dP/dQ curve will penetrate the upper pink region.

In this work, we propose a novel technique, namely differential pressure sensing (DPS) which measures the change of cell pressure per unit of charge (dP/dQ), to demonstrate an operando nondestructive strategy to detect the Li-plating event in multilayer pouch cells (Fig. 1a and Supplementary Fig. 1). We utilize an accessible external pressure sensor that does not demand modifying the existing battery’s internal configuration and manufacturing process. By measuring the real-time change of (dP/dQ) and comparing it with the threshold defined by the maximum of dP/dQ during intercalation, the Li-plating before its extensive growth can be captured with high precision. We also demonstrate that this strategy can realize a self-modulated charging protocol to avoid catastrophic Li-plating triggered by varied environmental conditions. This method only uses a single numerical threshold as a binary classifier to distinguish Li-plating and Li-ion intercalation, which poses minimal computational stress to the BMS and therefore shows great potential for future onboard integration.

## Results

### Principle of DPS for Li-plating detection

Electrode materials expand/shrink during battery cycling. When a cell is charged, the graphite anode expands ~13.1% in volume (4.2% in thickness) while the lithium nickel manganese cobalt oxide (NMC) and lithium cobalt oxide cathode materials contract for 3% and 1% in volume (1% and 0.3% in thickness), respectively^24,25^. LIB is a confined-volume system where the cell core is usually mechanically constrained by a solid casing. Therefore, the volume changes in the battery materials will eventually translate to the pressure change (ΔP) against the wall of the constraints. Since graphite anode thickness changing significantly exceeds the common cathodes, the full-cell pressure is inherently dominated by the graphite anode^24^ and can potentially reveal the electrochemical process on the anode.

During Li-plating, Li metal preferentially deposits on the top surface of the anode instead of utilizing the interplanar spacing within the graphite lattice (Supplementary Fig. 2)^26^. Consequently, for the same amount of Li^+^ ions, Li-plating causes a much more significant thickness/pressure increase than intercalation (Fig. 1b). For instance, the graphite anode used in this study has a coating thickness of 70 µm with an areal capacity of 2.84 mAh/cm^2^. Theoretically, its thickness only increases ~2 µm when the cell is fully charged. In contrast, if the same areal capacity is delivered by Li-plating in the dense form, the change of thickness will be at least ~14 µm without even considering the mossy structure of plated Li. Since the change in thickness leads to the change in pressure, Li-plating causes a much higher pressure change per unit of charge than intercalation, which can be described by the derivative of pressure to capacity as shown in Eq. (1), where P is the full-cell pressure and Q is the charge capacity. The maximum of dP/dQ during intercalation establishes a threshold to identify Li-plating: dP/dQ will remain under the threshold during intercalation but go beyond the threshold when Li-plating happens.

1 $${\left(\frac{{{{{{\rm{dP}}}}}}}{{{{{\rm{dQ}}}}}}\right)}_{{{{{\rm{plating}}}}}}\, > \,{\left(\frac{{{{{{\rm{dP}}}}}}}{{{{{\rm{dQ}}}}}}\right)}_{{{{{\rm{intercalation}}}}}}$$

To obtain the differential pressure dP/dQ, the operando cell pressure P is firstly measured on a lab-made 70 mAh 5-layer jelly-roll pouch cell consisting of graphite anode, NMC532 cathode, and 1 M LiPF_6_ in ethylene carbonate/diethyl carbonate (EC/DEC) electrolyte (Supplementary Fig. 1a). With a configuration similar to the previous report^24^, the cell is stacked with a metal force-distribution plate and a load cell before being clamped into a bench vise with a fixed thickness (Fig. 1a). A load cell is a common and cost-effective force sensor. The initial clamping pressure ranges from 20 to 50 psi which was reported to benefit the long-term cycling of graphite/NMC pouch cells^27^. The volume expansion of graphite is mainly due to the increment in the interplanar lattice distance after Li^+^ ion intercalation. To idealize the volume evolution, we plotted the average interplanar spacing of graphite lattice (Fig. 1c green curve) at different states of charge (SOC), as measured by X-ray diffraction (XRD)^28^. The average interplanar spacing of graphite lattice shows a similar trend of correlation with the measured operando pressure (orange curve in Fig. 1c) of the cell cycled at 0.2 C between 3 V and 4.2 V, which confirms that the graphite anode determines the pressure evolution of LIBs.

The differential pressure dP/dQ is then calculated and plotted in Fig. 1d. The absolute value of dQ is used to distinguish the charging period (dP/|dQ| is positive) and discharging charging period (dP/|dQ| is negative). As the charging starts, pristine graphite quickly transits to LiC_30_. This transition has the steepest slope of lattice expansion as shown in Fig. 1c, projecting the maximum of dP/dQ (0.815 psi/mAh at 6.4% SOC) which can be defined as the upper bound of Li-intercalation (red dashed line in Fig. 1d). When the anode undergoes Li-ion intercalation reactions, the dP/dQ curve would always stay within the blue region underneath the threshold. As the charging continues, dP/dQ declines to a minimum value (0.100 psi/mAh at 41.8% SOC) as the graphite goes through the transition from LiC_18_ to LiC_12_ during which the volume change is negligible. Following the minimum, dP/dQ rises again and reaches a large plateau in the entire latter half charging period due to the transition from LiC_12_ to LiC_6_ which contributes to about 50% of the cell capacity. As LIB is known as the rocking chair battery, the Li-ion intercalation reaction is highly reversible, and the discharge portion of dP/dQ profile is nearly symmetric to the charging portion. In contrast, when Li-plating happens, dP/dQ can be expected to go beyond the threshold established above and enter the upper Li-plating region labeled in Fig. 1d. In addition, the fact that the maximum of dP/dQ located at the beginning of charging (6.4% SOC) gives us a significant advantage in re-calibrating the system at any time. For example, the cell can be charged to 10% SOC under a slow charging rate to re-establish the threshold for Li-plating. It is worth mentioning that SEI or “dead lithium” buildup along cycling could potentially change the base pressure of the cell, they do not affect the derivative value of dP/dQ. Based on these working principles, DPS shows great recognition and sensitivity in identifying different anode electrochemical processes.

### DPS reveals Li-plating under the fast-charging condition

**Fig. 2:**
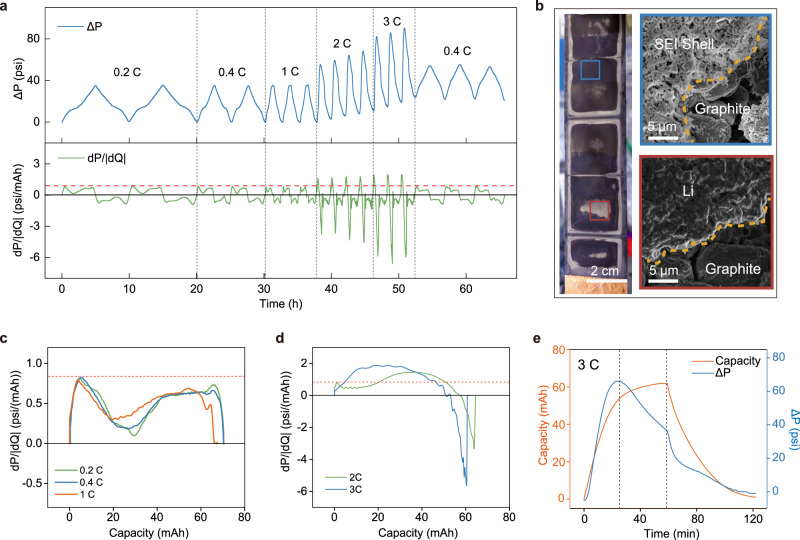
Differential pressure sensing reveals Li-plating during fast charging. **a** Pressure evolution of a 70 mAh Graphite/NMC532 multilayer pouch cell cycled at different C-rates (upper). The cell is charged and discharged symmetrically between 3 V and 4.2 V at different C-rates with a constant-current constant-voltage (CC-CV) charging protocol under a cut-off current of 0.1 C. The corresponding differential pressure profile (bottom) shows Li-plating is detected at 2 C and 3 C charging. **b** Optical (left) and SEM (right) images of the graphite anode after cycling. The blue and red squares show the regions with SEI shell and dead Li, respectively. **c** The dP/dQ profiles of the cell charged at different slow charging rates (≤1 C here) have similar maximums and stay below the defined threshold throughout the entire charging period. **d** The dP/dQ profiles of the cell charged at fast-charging C-rates (2 C and 3 C) go beyond the threshold indicating Li-plating. **e** The pressure change and charge capacity profiles at 3 C charge/discharge show that when there is Li-plating, the pressure reaches maximum before the capacity does. The misalignment of these two peaks causes the negative portion of the dP/dQ during charging.

To examine the dP/dQ response in the Li-plating event, the lab-made 70 mAh pouch cell is charged and discharged symmetrically between 3 V and 4.2 V at different C-rates with a constant-current constant-voltage (CC-CV) protocol and a cutoff current of 0.1 C. The operando pressure and the associated dP/dQ profiles versus time are presented in Fig. 2a. For slow charging rates such as 0.2 C, 0.4 C, and 1 C, the dP/dQ profile is enveloped underneath the red dashed line which is the Li-plating threshold defined by the maximum of dP/dQ at 0.2 C. Both the pressure and dP/dQ profiles during each charging/discharging cycle are highly symmetric indicating the reversible Li-intercalation reaction and the absence of Li-plating. In contrast, the symmetry breaks at 2 C and 3 C rates while the dP/dQ curve extends beyond the threshold as a result of Li-plating. In addition, both the maximal operando pressure and the pressure at the discharged state gradually increase for each cycle which could indicate SEI buildup and “dead Li” formation associated with Li-plating. Multiple studies have reported that the plated Li is only partially reversible, which can be reflected by CE in Supplementary Fig. 3^29–32^. While the reversible portion of the Li can migrate and re-intercalate into the graphite or is stripped during discharge, the irreversible Li electrically isolates from the anode, forming “dead Li”. Along with the excessive SEI growth due to the reaction between metallic Li and electrolyte, the “dead Li” adds irreversible thickness to the anode which causes a residual increase in pressure after each cycle. It is also noted that the “dead Li” cannot be retrieved by slower charge/discharge cycles, as the baseline pressure does not recover in the 0.4 C cycles after the fast charging. This increasing pressure does not change the dP/dQ features since an absolute change in pressure is not captured by the derivative, as discussed above. To visually confirm Li-plating at high C-rates, the discharged cell was disassembled in an argon-filled glovebox for post-cycling analysis. Figure 2b presents the optical and scanning electron microscopy (SEM) images taken of the graphite anode after cycling (discharged state). On the anode surface, the center and edges of each layer are covered by gray “dead Li”, displaying a compact morphology (red square in Fig. 2b) which is typical for Li metal deposition under pressure^33^. The dark area is covered by a layer of porous film which consists of empty SEI shells after most of the Li deposits are successfully extracted through discharge (blue square in Fig. 2b).

To better understand the characteristics of DPS, the dP/dQ profiles of each C-rate during charging are plotted against the charge capacity (Fig. 2c, d). Figure 2c shows that at 0.2 C, 0.4 C, and 1 C, all the dP/dQ profiles share identical maximum dP/dQ value and have similar behaviors. This phenomenon carries a significant value in practical application: a critical dP/dQ value obtained from a single dP/dQ profile measured at a sufficiently low charging rate can serve as a valid Li-plating threshold for various practical C-rates. However, the heterogeneity of lithiation across the thickness of the graphite anode can skew the dP/dQ profile. As a result, small variations of the dP/dQ profiles under different C-rates without Li-plating can be observed in Fig. 2c.

For the high charging rates which trigger Li-plating, the associated dP/dQ profiles for different rates are diverse due to the current-dependent Li-plating behavior. As shown in Fig. 2d, charging at 3 C reaches the threshold before 2 C and has a higher maximal value in dP/dQ. This indicates that Li-plating under 3 C not only happens earlier but also is more severe compared to 2 C. Another distinct feature of 2 C and 3 C’s dP/dQ profiles is the negative portion near the end of charging. In the Li-plating event, two electrochemical processes happen simultaneously: Li-plating and the migration of Li^+^ ions from the metallic Li into the graphite^19^. The first process is supplied by the charging current to expand the volume of Li deposits while the latter reduces the volume. With a high charging current, Li-plating exceeds the migration into graphite therefore the net volume of the metallic Li expands, increasing the cell pressure. However, when it is close to the end of the charging period, the cell is charged with constant voltage and the current gradually decreases to a point where Li-plating stops or is slower than the migration. Therefore, the net volume of the Li metal shrinks, causing the cell pressure to drop and dP/dQ to become negative before the charging is completed (Fig. 2e). The decline of pressure caused by migration is also measured by holding a cell at the charged state after Li-plating. It shows the migration can last for ~4 hours as the cell pressure keeps dropping before it finally stabilizes (Supplementary Fig. 4).

It is reasonable that the increased maximum cell pressure (or thickness) and the misaligned capacity/pressure peaks may also serve as fingerprints of Li-plating^20,21^; however, neither of them can resolve the Li-plating at an early stage because they can only detect Li-plating when the pressure reaches its peak during the constant-voltage charging region. But Li-plating could have already started much earlier. For example, the peak of the pressure at the 3 C case (Fig. 2e) is located at a late-charging stage where the cell is already charged to 53.9 mAh or 87.2% SOC of the full capacity (61.8 mAh) attainable at 3 C. In comparison, the dP/dQ curve passes the Li-plating threshold already when the cell is only charged to 5 mAh or 8.1% SOC. This difference highlights the sensitivity and capability of DPS for the early detection of Li-plating.

### Sensitivity and detection limit of DPS

**Fig. 3:**
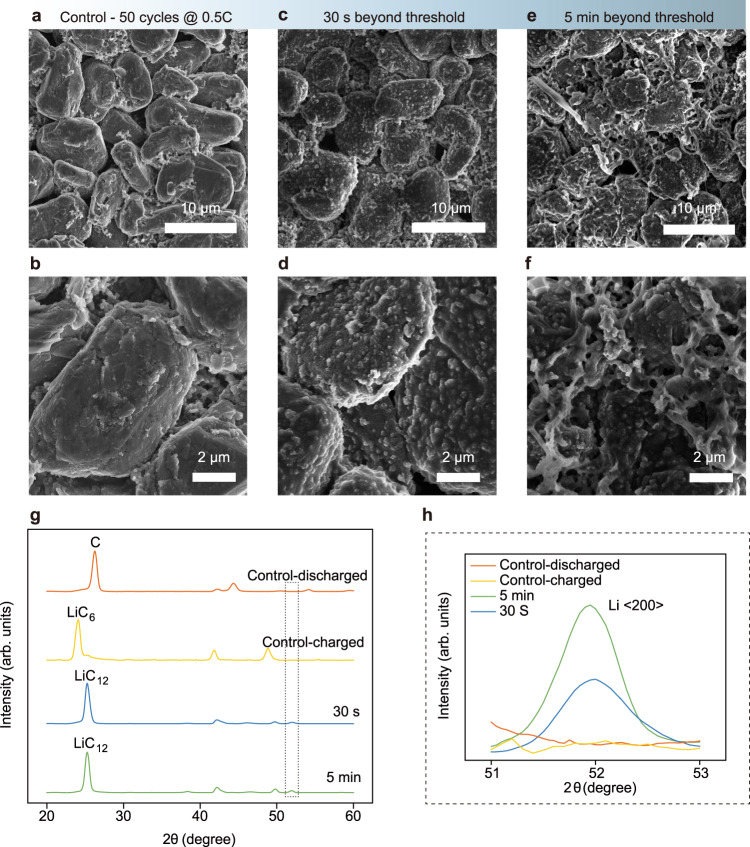
Surface morphology and XRD patterns of the graphite anode before and after dP/dQ passes the Li-plating threshold. **a**–**f** SEM images of the top surface of graphite anodes: Control sample which was cycled at 0.5 C for 50 cycles without fast charging (**a**, **b**); fast charging samples at 2 C terminated at 30 s (**c**, **d**) and 5 min (**e**, **f**) after dP/dQ goes beyond the Li-plating threshold, respectively. **g** The XRD patterns of control samples and fast-charged graphite anode samples. Both 30 s and 5 min samples resolve a mixture of partially lithiated graphite and metallic Li while the control samples only consist of pure graphite (discharged sample) or lithiated graphite (charged sample). **h** The enlarged XRD spectra near 51.97° shows the Li (200) peak intensity increases as the Li-plating continues.

To cross-validate the sensitivity and detection limit of the proposed dP/dQ method, the surface morphology and the composition of the graphite anode were examined with SEM and XRD. Three graphite samples were collected under different conditions: cycled at a low rate (0.5 C) without fast charging, cycled at a high rate (2 C) with fast charging after 30 s, and 5 min of continuous charging after dP/dQ passing the Li-plating threshold. After formation, the control sample which was cycled at 0.5 C for 50 cycles without fast charging has a smooth surface showing the native graphite SEI (Fig. 3a, b). In contrast, two cells were firstly cycled at 0.5 C to establish the Li-plating threshold followed by a high-rate (2 C) charging. The fast charging was terminated 30 s and 5 min after the real-time dP/dQ exceeded the threshold, respectively (Supplementary Fig. 5). Then the cells were immediately transferred into an argon-filled glovebox and disassembled once the testing was stopped for characterization. SEM images of the 30 s sample in Fig. 3c, d show that Li metal nucleates into small nanoparticles covering the surface of graphite particles which indicates the nanoparticle structure is not native graphite SEI but correlated to the fast-charging process. In the 5 min sample, though some of the nanoparticles are still observable, typical mossy lithium dendrites have formed on the surface of the graphite anode (Fig. 3e, f). The plating of lithium metal is also supported by XRD. For both samples that were charged at a fast rate, the associated XRD spectrum presented in Fig. 3g resolves a mixture of partially lithiated graphite (LiC_12_) and Li metal. In comparison, the control samples only consist of pure graphite when discharged or lithiated graphite (LiC_6_) when fully charged^28,34^. The scans near 51.97° are enlarged and replotted in Fig. 3h to show that while in the control samples no metallic Li can be detected, the Li (200) peak can be easily identified in the 30 s sample and its intensity increases as the fast charging continues which can be seen in the 5 min sample.

Combining SEM and XRD, we have elucidated that DPS can detect the Li-plating as early as the nucleation stage. It also reveals that Li-plating is initiated by the nucleation of small Li nanoparticles before forming dendrites which agree with the recent discovery of the morphological evolution of the electrochemically plated Li under stress^35^. It is reported that the Li metal starts with the sluggish nucleation of Li nanoparticles. Then the spherical particle slowly expands until the dendrite sprouts of the interphase between Li particle and electrolyte, leading to a rapid anisotropic growth along the longitudinal direction of the dendrite. Because the “dead Li” formation and the major safety concerns of Li-plating are direct results of the dendritic structure^36^, being able to detect Li-plating before the rapid catastrophic dendritic growth provides us a buffer to dynamically modulate the charging current so that subsequent dendritic growth can be terminated.

### Self-regulated charging enabled by detection of Li-plating with dP/dQ

Developing a fast-charging protocol remains challenging, given many factors that rate performance is related to, such as the environment where the batteries are being used, the state of degradation, the heterogeneities within a cell, etc. This challenge is even more pronounced in the context of EVs, considering the wide span of consumers’ driving habits and geographical distribution which are all coupled to different cell degradation modes. The large parameter space makes it difficult to develop a ‘rigid’ universal fast-charging protocol that is compatible with all conditions and the entire cell lifespan. Currently, it is typical that battery companies only provide a few preset charging protocols which are rigid, reflecting little on the environmental variables and the state of degradation^37^. It could eventually trigger Li-plating when variables change and result in rapid cell degradation.

**Fig. 4:**
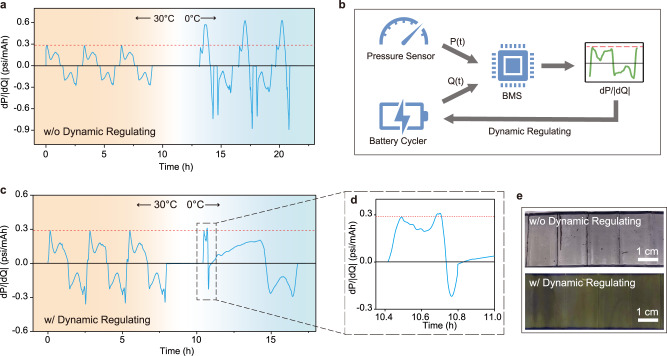
Dynamic charging regulated by dP/dQ avoids catastrophic Li-plating under low temperatures. **a** 200 mAh commercial battery with rolled graphite/NMC532 electrodes cycled at 1 C under 30 °C and 0 °C without dynamic regulation, dP/dQ displays Li-plating triggered by low temperature (0 °C). **b** Scheme for self-regulated charging. BMS calculates and monitors the real-time dP/dQ value, when it senses Li-plating, the current is modulated to extinguish the plating. **c**, **d** The 200 mAh commercial cell is cycled at 1 C under 30° and 0° with the dynamic regulation of BMS, Li-plating is effectively contained. **e** Optical images of the cycled graphite anodes at the charged state. Without dynamic regulation, the anode surface is covered by a thick layer of Li metal; with dynamic regulating, the anode shows a golden color, known as lithiated graphite in LiC_6_ phase.

To demonstrate the incompatibility of a rigid charging protocol, we cycled a commercial 200 mAh pouch cell with rolled graphite/NMC532 electrodes at different controlled temperatures in an environmental chamber, using a 1 C CC-CV charging protocol with a cut-off current of 0.1 C. As shown in Fig. 4a, the cell cycles normally at 30 °C without Li-plating as the dP/dQ profile is contained by the threshold. However, after the temperature decreases to 0 °C, the same charging protocol triggers severe Li-plating which can be concluded from the elevated dP/dQ value. The cell is then disassembled at the charged state after 3 cycles at 0 °C, revealing that the entire graphite anode is covered by a thick layer of Li metal (Fig. 4e optical image, top, gray color as an indicator of Li metal).

One emerging application for the onboard detection of Li-plating is providing a solution to the insufficient rigid conventional charging protocol. By calculating the real-time dP/dQ value and then comparing it against the numerical Li-plating threshold, the detection of Li-plating backed by DPS could be easily integrated into any embedded system such as BMS. With this added feature, BMS can dynamically regulate the charging current so that Li-plating can be terminated at its nucleation stage. As a proof of concept, we develop a Python program to simulate a BMS that retrieves data streams from both load cell and the battery cycler to calculate the dP/dQ value in real-time then dynamically regulate the charging current below the dP/dQ threshold (Fig. 4b). In this specific case, if the dP/dQ value exceeds the Li-plating threshold, the dynamic charge regulation kicks in, sending a signal to the battery cycler to turn the current to a safer and slower rate of 0.2 C. With this updated BMS, the same type of commercial cell is cycled again under the same condition with the same 1 C charging protocol as shown in Fig. 4c. At 30 °C, the dynamic regulation does not interfere with the charging because Li-plating is not detected. At 0 °C, dynamic regulation is triggered to reduce the charging current immediately after the dP/dQ value exceeds the threshold due to Li nucleation. After dynamic regulation takes control, the dP/dQ value drops below the threshold immediately indicating Li-plating is effectively extinguished (Fig. 4d). The cell is then opened at the charged state in glovebox after three dynamically regulated charging to show that the golden color of lithiated graphite in LiC_6_ phase (Fig. 4e, optical image, bottom) without a sign of Li-plating is observed^24,38^, indicating that self-regulated charging can effectively contain Li-plating under an unexpected harsh charging condition.

## Discussion

Unlike traditional large and specialized equipment for detecting Li-plating in laboratory research, the pressure sensor demonstrated here shows good potential in the integration with BMS for onboard application. As a battery pack structure can be divided into modules, we can homogeneously locate the sensors in different places and balance them by BMS. Compared to a single cell, the battery module brings a more pronounced response to pressure changes during fast charging, which can further increase the sensitivity of DPS in practical applications.

In summary, we demonstrate DPS, which measures the pressure change per unit of charge in the battery, as a possible tool to distinguish the electrochemical processes of lithium-ion intercalation and Li plating. This technique utilizes an accessible external force sensor and maintains the existing battery’s internal configuration, showing high precision in detecting Li-plating events. With the dP/dQ threshold established by the intercalation reaction of slow charging, we successfully capture the Li-plating before its excessive growth in real-time under strenuous charging conditions. As a proof of concept, we show that with this integration, a dynamic-regulated charging protocol can be realized to effectively extinguish Li-plating triggered by low temperature (0 °C). This advanced and nondestructive technological approach promises to accelerate the development of fast-charging batteries and could inspire more reliable and safer battery designs.

## Methods

### Cell fabrication

Both lab-made and commercial cells are used in this study.

Nominal 200 mAh Li[Ni_0.5_Mn_0.3_Co_0.2_]O_2_ (NMC532)/graphite 402035-size jelly-roll dry commercial cells were purchased from Li-fun technology. The dry cells were vacuum-sealed during transportation and re-opened in an argon-filled glovebox (O_2_ and H_2_O levels are both less than 0.1 ppm) upon receiving. 700 µL 1 M LiPF_6_ in EC/DEC (1/1 by volume) electrolyte was filled into the cell followed by vacuum sealing.

For the 70 mAh lab-made cell, single-sided NMC532 and graphite electrodes were fabricated in the CAMP facility of Argonne National Laboratory with area capacities of 2.68 mAh/cm^2^ and 2.84 mAh/cm^2^, respectively. NMC532 (90% NMC, 5% super P carbon, and 5% PVDF binder) coated on aluminum foil was used as NMC cathode. Graphite (91.83% graphite, 2% super P carbon, 6% PVDF binder, and 0.17 % oxalic acid) coated on copper foil was used as graphite anode. Both cathode and anode were calendared before receiving. The electrodes were dried in a vacuum oven for 24 hours before cell making. The NMC532 cathode and graphite anode were cut into 1-inch-wide strips and then sandwiched with a 25 µm thick polyolefin separator (Celgard 2325) before being folded into 1-inch by 1-inch sized cell core in a zigzag manner (5-layer cathode, 5-layer anode, and 5-layer separator in total). The cell core is then sealed into an aluminum laminated pouch cell case with 500 µL 1 M LiPF_6_ in EC/DEC electrolyte. Both commercial cells and lab-made cells were allowed to rest for 24 hours or longer before a 0.1 C charging/discharging formation cycle between 3 V and 4.2 V in the ambient (about 25 °C) unless otherwise specified.

### Pressure measurement

A metal block (length × width × height: 1.2 inch × 1.2 inch × 1 inch) with a size slightly larger than the cell core was attached to the cell as a force-distribution plate. A button-style load cell (LBC-500, Transducer Techniques) was attached to the metal block. The entire stack was then clamped into a bench vise with an initial pressure ranging from 20 to 50 psi. The cell was allowed to rest for at least 12 hours to allow the materials to relax under compressive force before cycling. Each pouch cell contains a gasbag as shown in Supplementary Fig. 1 to avoid any generated gas effect. If any gas is generated during battery operation, it will be pushed into the gasbag due to the compressive force applied to the cell. Thus, the pressure measurement was not interfered with by any gas generation. We did not observe any significant gas generation during battery testing. Once the testing was stopped, the pouch cells were immediately in an argon-filled glovebox and then disassembled by cutting the two tabs one by one to avoid battery shorting during the disassembly process. This process takes ~5~10 minutes. Different initial stack pressures in our testing affect the maximum dP/dQ threshold but do not change the characteristics of the dP/dQ curves for Li-plating detection.

### Characterizations

SEM images were taken in NOVA and Magellan SEMs. Once the cells were disassembled, the electrode samples were washed with 1,2-dimethoxyethane (anhydrous, 99.5%, Millipore Sigma) by three times. Subsequently, the samples were stuck on the SEM stage in an argon-filled glovebox and sealed by an air-free container before being transferred into the SEM chamber. Samples were exposed to air for less than 5 s during the sample transfer process. XRD patterns of the graphite anode were recorded on a PANalytical X’Pert instrument. The XRD samples were peeled off from the Cu current collector and then sealed with Kapton tape in the argon-filled glovebox beforehand to protect them from the air.

## Supplementary information

Supplementary Information

## Data Availability

The relevant datasets generated and analyzed in this study are provided with this paper. Source data are provided with this paper.

## Source data

Source Data

## Code availability

The code for dynamic regulating is available from the corresponding author upon request.

## References

[1] Bibra, E. M. et al. Global EV Outlook 2021: Accelerating Ambitions Despite the Pandemic. (2021).

[2] IEA (2022), G. E. O., IEA, Paris, https://www.iea.org/reports/global-ev-outlook-2022 (2022).

[3] Knupfer, S. M. et al. Electrifying insights: how automakers can drive electrified vehicle sales and profitability. *McKinsey & Company* (2017).

[4] Tomaszewska A (2019). Lithium-ion battery fast charging: a review. eTransportation.

[5] Howel, D. et al. Enabling fast charging: a technology gap assessment. No. INL/EXT-17-41638 (US Department of Energy, 2017).

[6] Gallagher KG, Dees DW, Jansen AN, Abraham DP, Kang S-H (2012). A volume averaged approach to the numerical modeling of phase-transition intercalation electrodes presented for LixC6. J. Electrochem. Soc..

[7] Chu Z (2018). Testing lithium-ion battery with the internal reference electrode: an insight into the blocking effect. J. Electrochem. Soc..

[8] Liu Y, Zhu Y, Cui Y (2019). Challenges and opportunities towards fast-charging battery materials. Nat. Energy.

[9] Wang Q (2012). Thermal runaway caused fire and explosion of lithium ion battery. J. Power Sources.

[10] Ye Y (2020). Ultralight and fire-extinguishing current collectors for high-energy and high-safety lithium-ion batteries. Nat. Energy.

[11] Lin D, Liu Y, Cui Y (2017). Reviving the lithium metal anode for high-energy batteries. Nat. Nanotechnol..

[12] Zhang SS (2006). The effect of the charging protocol on the cycle life of a Li-ion battery. J. Power Sources.

[13] Keil P, Jossen A (2016). Charging protocols for lithium-ion batteries and their impact on cycle life—an experimental study with different 18650 high-power cells. J. Energy Storage.

[14] Burns J, Stevens D, Dahn J (2015). In-situ detection of lithium plating using high precision coulometry. J. Electrochem. Soc..

[15] Downie L (2013). In situ detection of lithium plating on graphite electrodes by electrochemical calorimetry. J. Electrochem. Soc..

[16] Jin Y (2020). Detection of micro-scale Li dendrite via H2 gas capture for early safety warning. Joule.

[17] Campbell ID, Marzook M, Marinescu M, Offer GJ (2019). How observable is lithium plating? Differential voltage analysis to identify and quantify lithium plating following fast charging of cold lithium-ion batteries. J. Electrochem. Soc..

[18] Konz ZM, McShane EJ, McCloskey BD (2020). Detecting the onset of lithium plating and monitoring fast charging performance with voltage relaxation. ACS Energy Lett..

[19] Schindler S, Bauer M, Petzl M, Danzer MA (2016). Voltage relaxation and impedance spectroscopy as in-operando methods for the detection of lithium plating on graphitic anodes in commercial lithium-ion cells. J. Power Sources.

[20] Spingler FB, Wittmann W, Sturm J, Rieger B, Jossen A (2018). Optimum fast charging of lithium-ion pouch cells based on local volume expansion criteria. J. Power Sources.

[21] Bitzer B, Gruhle A (2014). A new method for detecting lithium plating by measuring the cell thickness. J. Power Sources.

[22] Samad NA, Kim Y, Siegel JB, Stefanopoulou AG (2016). Battery capacity fading estimation using a force-based incremental capacity analysis. J. Electrochem. Soc..

[23] Oh K-Y, Epureanu BI, Siegel JB, Stefanopoulou AG (2016). Phenomenological force and swelling models for rechargeable lithium-ion battery cells. J. Power Sources.

[24] Louli A, Ellis L, Dahn J (2019). Operando pressure measurements reveal solid electrolyte interphase growth to rank Li-Ion cell performance. Joule.

[25] Koyama Y (2006). Harnessing the actuation potential of solid‐state intercalation compounds. Adv. Funct. Mater..

[26] Wang C, Ma Z, Wang Y, Lu C (2016). Failure prediction of high-capacity electrode materials in lithium-ion batteries. J. Electrochem. Soc..

[27] Cannarella J, Arnold CB (2014). Stress evolution and capacity fade in constrained lithium-ion pouch cells. J. Power Sources.

[28] Missyul A, Bolshakov I, Shpanchenko R (2017). XRD study of phase transformations in lithiated graphite anodes by Rietveld method. Powder Diffr..

[29] Ren D (2018). Investigation of lithium plating-stripping process in Li-ion batteries at low temperature using an electrochemical model. J. Electrochem. Soc..

[30] Wandt J, Jakes P, Granwehr J, Eichel R-A, Gasteiger HA (2018). Quantitative and time-resolved detection of lithium plating on graphite anodes in lithium ion batteries. Mater. Today.

[31] Paul PP (2021). Quantification of heterogeneous, irreversible lithium plating in extreme fast charging of lithium-ion batteries. Energy Environ. Sci..

[32] Liu F (2021). Dynamic spatial progression of isolated lithium during battery operations. Nature.

[33] Yin X (2018). Insights into morphological evolution and cycling behaviour of lithium metal anode under mechanical pressure. Nano Energy.

[34] Shi F (2017). Strong texturing of lithium metal in batteries. Proc. Natl Acad. Sci. USA.

[35] He Y (2019). Origin of lithium whisker formation and growth under stress. Nat. Nanotechnol..

[36] Li Y (2018). Correlating structure and function of battery interphases at atomic resolution using cryoelectron microscopy. Joule.

[37] Duru KK (2021). Critical insights into fast charging techniques for lithium-ion batteries in electric vehicles. IEEE Trans. Device Mater. Reliab..

[38] Maire P, Evans A, Kaiser H, Scheifele W, Novák P (2008). Colorimetric determination of lithium content in electrodes of lithium-ion batteries. J. Electrochem. Soc..

