# Supplementary material for: Onboard early detection and mitigation of lithium plating in fast-charging batteries
Source: Nat Commun. 2022 Nov 19;13:7091. doi: 10.1038/s41467-022-33486-4 (PMC9675798; doi:10.1038/s41467-022-33486-4)
Supplement: Supplementary file 1 — Supplementary Information [file 41467_2022_33486_MOESM1_ESM.pdf]

Supplementary Information for

Onboard Early Detection and Mitigation of Lithium Plating in Fast-Charging Batteries

Wenxiao Huang[1,#], Yusheng Ye[1,#], Hao Chen[1], Rafael A. Vilá[1], Andrew Xiang[2], Hongxia Wang[1], Fang Liu[1], Zhiao Yu[3], Jinwei Xu[1], Zewen Zhang[1], Rong Xu[1], Yecun Wu[4], Lien-Yang Chou[1], Hansen Wang[1], Junwei Xu[5], David Tomas Boyle[1], Yuzhang Li[1,6*] & Yi Cui [1,5*]

Affiliations

1. Department of Materials Science and Engineering, Stanford University, Stanford, CA 94305, USA.
2. College of Letters and Science, University of California, Berkeley, CA 94720, USA.
3. Department of Chemical Engineering, Stanford University, Stanford, CA 94305, USA.
4. Department of Electrical Engineering, Stanford University, Stanford, CA 94305, USA.
5. SLAC National Accelerator Laboratory, Stanford Institute for Materials and Energy Sciences, Menlo Park, CA 94025, USA.
6. Chemical and Biomolecular Engineering, University of California, Los Angeles, CA 90095, USA.

# These authors contributed equally to this work.

* Correspondence and requests for materials should be addressed to:

Yuzhang Li (Email: yuzhangli@ucla.edu)

Yi Cui (Email: yicui@stanford.edu).

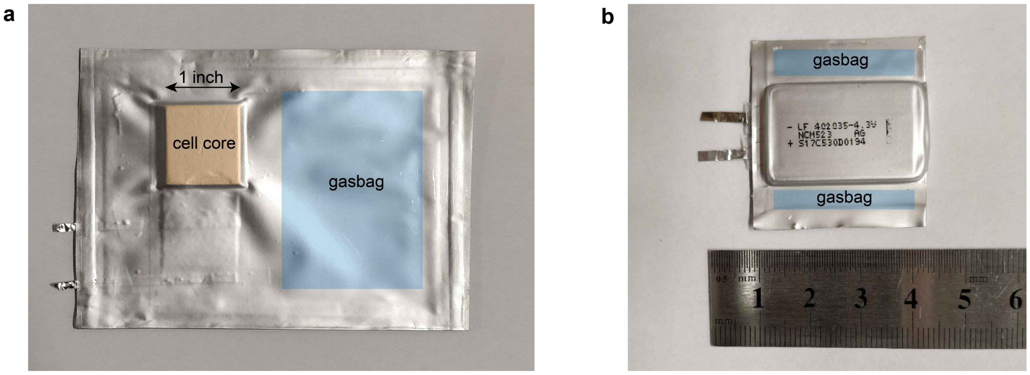

Supplementary Figure 1. Picture of pouch cells with gasbags. (A) Lab-made 70 mAh multilayer NMC532/graphite pouch cell with a core size of 1 inch by 1 inch. (B) Commercial cell with 200 mAh capacity and artificial NMC532/graphite electrodes. Both cells contain a gasbag (blue area) to avoid gas generation interference with pressure measurement.

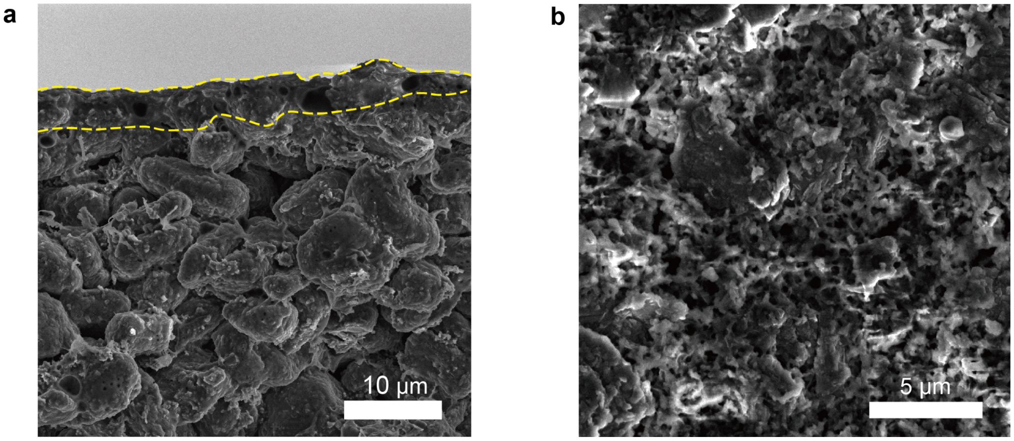

Supplementary Figure 2. SEM of the cross-section and top surface of graphite electrode with Li-plating. A lab-made cell was charged to 4.2 V at 3 C to trigger Li-plating. (A) The cross-section of the graphite electrodes shows that though Li nucleation can be observed on the surface of graphite particles across the entire electrode's thickness, the majority of the Li-plating happens on the top surface of the electrode instead of utilizing the void space between the graphite particles, which induces significant thickness change to the electrodes. (B) The top view of the electrode shows mossy metallic lithium covering the surface of the graphite electrode.

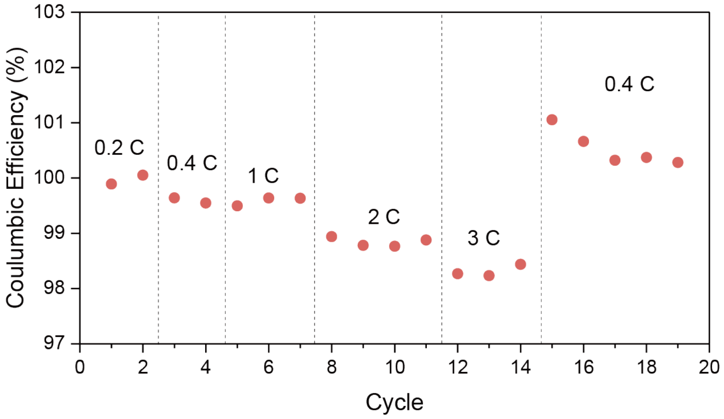

Supplementary Figure 3. Coulombic efficiency (CE) of the cell at different C-rates. CE of the cycling is displayed in Figure 2 of the main text. It shows that CE immediately decreases in high-rate cycles such as 2 C and 3 C, where Li-plating happens and removes Li inventory in the ways of dead Li and excessive SEI formation. The 0.4 C cycle after 3 C charging shows CE greater than 100% which indicates part of the plated metallic Li is reversible. Two possible pathways could lead to this reversibility: 1. The graphite particles are pre-lithiated by the plated metallic Li before 0.4 C charging; 2. Part of the plated Li is extracted through discharge of the 0.4 C cycles.

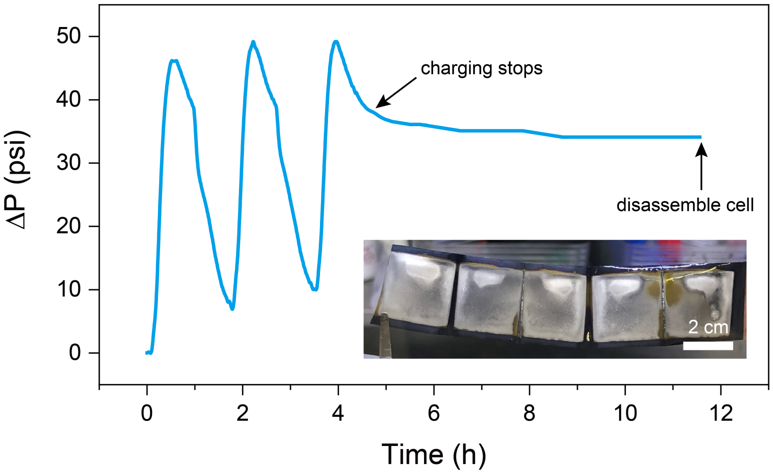

Supplementary Figure 4. Pressure profile of the Li-plated cell being held at the charged state. After the same cycling shown in Figure 2, three extra 2 C charges were applied to a lab-made cell to assure Li-plating, the cell was then held at the charged state. The pressure profile shows that after the cycling stops, the pressure keeps decreasing then stabilizes after 8 hours. The pressure drop after charging indicates the plated metallic Li continuously diffuses then re-intercalates into graphite particles until only dead Li remains. The inset optical image shows the dead Li on the surface of graphite after the experiment.

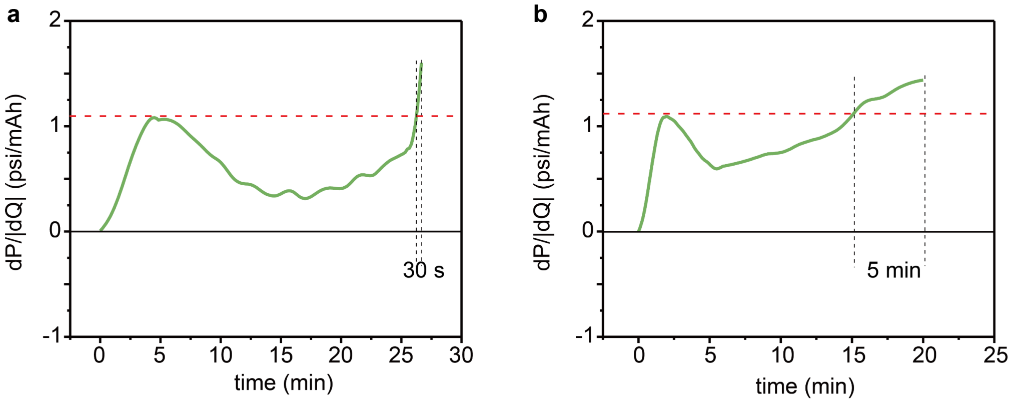

Supplementary Figure 5. The dP/dQ profile of the cells used for Li-plating morphology study. The dP/dQ profiles of the cells are displayed in Figure 2 of the main text. The two cells were disassembled for SEM imaging 30 s (a) and 5 min (b) respectively after dP/dQ passes the Li-plating threshold.
